# Methylmercury Causes Blood-Brain Barrier Damage in Rats via Upregulation of Vascular Endothelial Growth Factor Expression

**DOI:** 10.1371/journal.pone.0170623

**Published:** 2017-01-24

**Authors:** Tetsuya Takahashi, Masatake Fujimura, Misaki Koyama, Masato Kanazawa, Fusako Usuki, Masatoyo Nishizawa, Takayoshi Shimohata

**Affiliations:** 1 Department of Neurology, Brain Research Institute, Niigata University, Niigata, Niigata, Japan; 2 Department of Basic Medical Sciences, National Institute for Minamata Disease, Minamata, Kumamoto, Japan; 3 Department of Clinical Medicine, National Institute for Minamata Disease, Minamata, Kumamoto, Japan; Hungarian Academy of Sciences, HUNGARY

## Abstract

Clinical manifestations of methylmercury (MeHg) intoxication include cerebellar ataxia, concentric constriction of visual fields, and sensory and auditory disturbances. The symptoms depend on the site of MeHg damage, such as the cerebellum and occipital lobes. However, the underlying mechanism of MeHg-induced tissue vulnerability remains to be elucidated. In the present study, we used a rat model of subacute MeHg intoxication to investigate possible MeHg-induced blood-brain barrier (BBB) damage. The model was established by exposing the rats to 20-ppm MeHg for up to 4 weeks; the rats exhibited severe cerebellar pathological changes, although there were no significant differences in mercury content among the different brain regions. BBB damage in the cerebellum after MeHg exposure was confirmed based on extravasation of endogenous immunoglobulin G (IgG) and decreased expression of rat endothelial cell antigen-1. Furthermore, expression of vascular endothelial growth factor (VEGF), a potent angiogenic growth factor, increased markedly in the cerebellum and mildly in the occipital lobe following MeHg exposure. VEGF expression was detected mainly in astrocytes of the BBB. Intravenous administration of anti-VEGF neutralizing antibody mildly reduced the rate of hind-limb crossing signs observed in MeHg-exposed rats. In conclusion, we demonstrated for the first time that MeHg induces BBB damage via upregulation of VEGF expression at the BBB *in vivo*. Further studies are required in order to determine whether treatment targeted at VEGF can ameliorate MeHg-induced toxicity.

## Introduction

Methylmercury (MeHg) is a by-product formed during acetaldehyde synthesis. MeHg also occurs in nature due to the microbial methylation of mercury. Artificially produced MeHg has caused serious environmental problems over the past 60 years in Japan [1],[2]. Although extensive artificial MeHg pollution has been reduced, the naturally occurring environmental form is increasing due to increasing mercury emission into the atmosphere associated with human activities [3]. At toxic exposure levels, MeHg causes central nervous system insults such as Hunter-Russell syndrome [4], Minamata disease [5],[6], and Niigata Minamata disease [7]. Clinical manifestations of MeHg intoxication in adults include cerebellar ataxia, concentric constriction of visual fields, and sensory and auditory disturbances. The specific symptoms depend on the location of the lesions induced by MeHg, which often produce damage to the cerebellum and occipital lobes [8]. However, the underlying mechanism of MeHg-induced tissue vulnerability remains to be elucidated.

Recent studies suggest that certain factors such as oxidative stress and blood-brain barrier (BBB) damage may modify MeHg-induced neurotoxicity. We previously demonstrated that anti-oxidative compounds prevent MeHg-induced neurotoxicity in rats [9],[10], and that decreased expression of anti-oxidative enzymes enhances the vulnerability of cerebellar granular cells to MeHg toxicity [11]. This observation suggests a crucial role of oxidative stress in MeHg-induced neuronal toxicity. Furthermore, a recent study demonstrated that MeHg induces the expression of vascular endothelial growth factor (VEGF), a potent angiogenic growth factor, in cultured endothelial cells [12]. This report prompted us to investigate whether MeHg causes BBB damage by inducing VEGF expression *in vivo*, as increased VEGF expression results in BBB damage that leads to hemorrhage, edema, and microcirculation failure in a number of conditions [13]. In the present study, we utilized a rat model of subacute MeHg intoxication to investigate whether MeHg produces BBB damage via upregulation of VEGF expression.

## Materials and Methods

### Rat model of subacute MeHg intoxication

**Fig 1:**
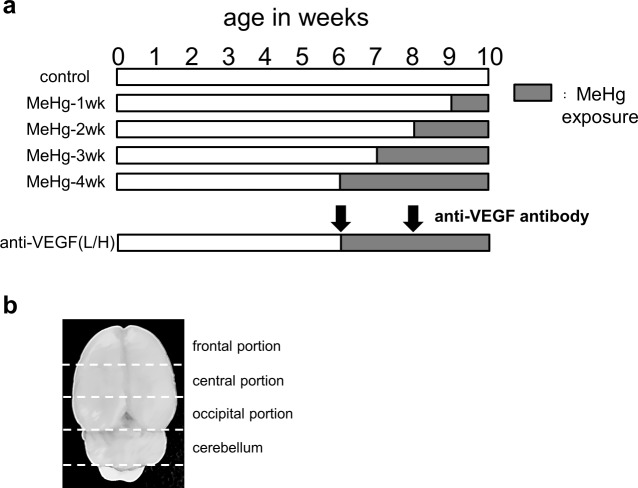
Time course of experiments and sample preparation. (a) Six-week-old male Wistar rats were divided into five groups as follows: control, 1-, 2-, 3-, and 4-week MeHg exposure groups (n = six per group). Rats were administered water with or without 20-ppm MeHg from 6 weeks of age. Rabbit anti-VEGF neutralizing antibody was intravenously administered twice at 6 and 8 weeks of age (arrow) at doses of 10 and 20 μg (42–44 and 56–64 μg/kg), respectively, for the low-dose anti-VEGF (L) group or 20 and 100 μg (81–87 and 301–332 μg/kg), respectively, for the high-dose anti-VEGF (H) group. Brain tissues were harvested at 10 weeks of age. (b) Brains were separated into the cerebellum and cerebrum; the cerebrum was further separated into frontal, central, and occipital regions.

The present study was carried out in strict accordance with the recommendations of the Guide for the Care and Use of Laboratory Animals of the National Institutes of Health. All experimental animal protocols were approved by the National Institute for Minamata Disease (Permit Number: 2012,7–1). Six-week-old male Wistar rats obtained from CLEA Japan (Tokyo, Japan) were housed two to three per cage at 23°C (±3°C) with humidity 55% (±15%) under a 12-h light-dark cycle. All rats were allowed free access to pelleted diet (CE-2, CLEA Japan) and tap water. The rats were divided into five groups as follows: control, 1-, 2-, 3-, and 4-week MeHg exposure groups (n = 6 per group). MeHg was administered as previously described [14]. Briefly, the rats were provided water with or without 20-ppm MeHg from 6 weeks to 9 weeks of age, depending on exposure group, until the end of the experiment at 10 weeks of age (Fig 1A). Average mercury intake for each rat was 1.0 mg∙kg^-1^∙day^-1^. In this condition, mercury content in the brain will be above 10 ppm after 3 weeks of exposure. It is thought that more than 10 ppm of mercury in the brain is required to cause brain injury in both humans and rats. Body weight was measured twice a week. Brain tissues were harvested at 10 weeks old (Fig 1A) and separated into the cerebellum and cerebral cortex, which was then further separated into three portions (frontal, central, and occipital; Fig 1B).

### Measurement of mercury content

The total concentration of mercury in each section of the brain tissue samples was determined according to the oxygen combustion-gold amalgamation method [15] using a mercury analyzer (MA2000, Nippon Instruments, Tokyo, Japan).

### Evaluation of neuronal impairment

The “hind-limb crossing sign,” a characteristic sign of MeHg intoxication [16], was used to evaluate the neuronal impairment of the rats and was determined at 10 weeks according to previously described protocols [17]. This sign is considered an expression of limb ataxia caused by impairments in deep sensation or injury to the cerebellum in animal models of MeHg intoxication. Briefly, rats were held by the tail, and the posture of both hind-limbs was observed. The level of neuronal impairment was evaluated using the following scale: (+++), limbs crossed each other; (++), limbs nearly crossed; (+), limbs did not cross and flexion was observed; and (-), limbs moved freely and were typically splayed outward.

### Immunohistochemistry

Brain tissue samples were prepared from rats at 10 weeks of age following deep anesthesia by overdose of isoflurane (Wako Pure Chemical Industries, Osaka, Japan). The rats were perfused with intracardiac cold saline followed by 4% paraformaldehyde in 0.1 M phosphate-buffered saline (PBS; pH 7.4). VEGF expression was then examined using immunohistochemical (IHC) staining with rabbit anti-VEGF antibody (A-20, Santa Cruz, CA, USA, 1:100). To investigate the cellular distribution of VEGF, IHC staining was performed using antibodies against rat endothelial cell antigen-1 (RECA-1)—an endothelial marker protein (MCA-970R, Serotec, Oxford, UK, 1:250)—and glial fibrillary acidic protein (GFAP), an astrocytic marker (#3670, Cell Signal Technology, Beverly, MA, USA, 1:50). The sections were mounted and examined with a fluorescence microscope (BZ-9000, Keyence, Japan). Horseradish peroxidase-conjugated anti-rat IgG antibody (PK-6104, VECTASTAIN Elite ABC Kit (Rat IgG), Vector Laboratories, CA, USA) was used to detect extravascular intrinsic serum IgG. All rats in each group were used for staining, and control tissue sections were run without primary antibody, which confirmed that there was no specific staining.

### Treatment with anti-VEGF neutralizing antibody

To assess the effect of VEGF in the MeHg intoxication model, we administered rabbit anti-VEGF neutralizing antibody (RB-222, Lab Vision-Neomarkers, Fremont, CA, USA), which has been shown to neutralize VEGF in rat brains [18],[19]. Rats were anesthetized and maintained under 2.5% isoflurane in air using a face mask. RB-222 was intravenously administered twice at 6 and 8 weeks of age at doses of 10 and 20 μg (42–44 and 56–64 ug/kg), respectively, for the low-dose anti-VEGF (L) group or 20 and 100 μg (81–87 and 301–332 ug/kg), respectively, for the high-dose anti-VEGF (H) group. The doses differed between weeks 6 and 8 due to the weight gain of the rats. Brain tissues were harvested at 10 weeks old (n = six to eight per group).

### Statistical analysis

The mercury content among four lesions were statistically compared using one-way ANOVA. Differences in the frequencies were assessed using Fisher’s exact test. Differences were considered significant at P < 0.05.

## Results

### Mercury content of brain tissue samples

**Fig 2:**
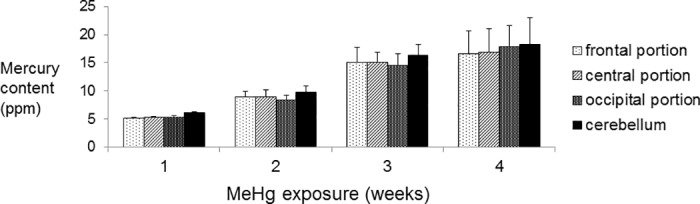
Mercury content in each region of brain based on duration of methylmercury exposure.

We first measured the mercury content of rat brains exposed to MeHg using the oxygen combustion-gold amalgamation method. Our analysis revealed that longer MeHg exposure resulted in higher mercury content in rat brains (Fig 2). No significant differences in mercury content were observed among the brain region samples in the same MeHg exposure duration group.

The mercury content of rat brain samples from various regions (frontal, central, and occipital regions, in addition to the cerebellum) from control or 1-, 2-, 3-, and 4-week MeHg exposure groups were measured using the oxygen combustion-gold amalgamation method with a mercury analyzer; n = five or six per group. The total concentration of mercury in the control group was less than 0.1 ppm in all tissues. No significant differences were observed in mercury content among the brain region samples in the same MeHg exposure duration group.

### Time course of changes in body weight

**Fig 3:**
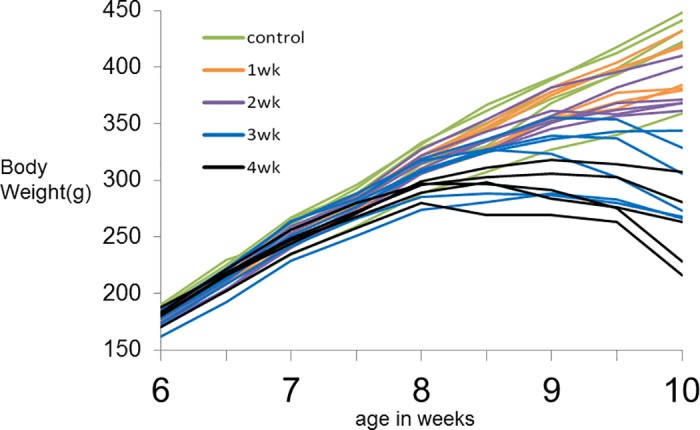
Time course of changes in body weight. Body weight was measured 2 times per week and plotted. Each line in the same color shown in the explanatory notes represents a single rat exposed to mercury in the same period. Body weight increased from 6 weeks to 10 weeks in the control group (green) and the 1- or 2-week exposure groups (orange and purple lines, respectively). However, body weight decreased following 2 or 3 weeks of exposure to methylmercury (after 8 weeks: black line, after 9 weeks: blue line).

The body weight of rats increased from 6 weeks to 10 weeks in the control group; however, the body weight of rats exposed to methylmercury decreased after 2 or 3 weeks of exposure (Fig 3).

### Hind-limb crossing sign after MeHg exposure

**Fig 4:**
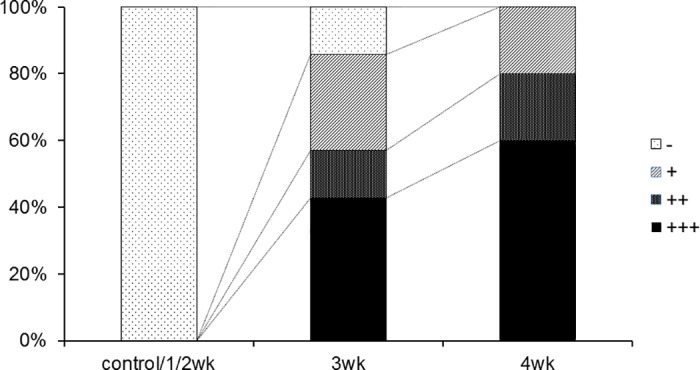
Incidence of hind-limb crossing signs after methylmercury exposure. Hind-limb crossing signs at 10 weeks of age are shown. Rats were held by the tail, and the posture of both hind-limbs was observed. This phenomenon was graded according to the following scale: (+++), limbs crossed each other; (++), limbs nearly crossed; (+), limbs did not cross and limb flexion was observed; and (-), limbs moved freely and were typically splayed outward. All the rat in control group and the rat in the 1- and 2-week MeHg exposure groups did not show a hind-limb crossing sign; therefore, the results for three groups are integrated into one bar (n = five or six per group).

The neuronal impairment of the rats was evaluated by assessing the presence of hind-limb crossing signs at 10 weeks of age (n = 6 per group, Fig 4). In the control, 1-, and 2-week exposure groups, no rats exhibited hind-limb crossing. However, in the 3-week exposure group, only one rat exhibited normal hindlimb movement. In the 4-week exposure group, all rats exhibited complete hind-limb crossing. These findings suggest that longer MeHg exposure is associated with increased neuronal impairment.

### VEGF expression in astrocytes following MeHg exposure

**Fig 5:**
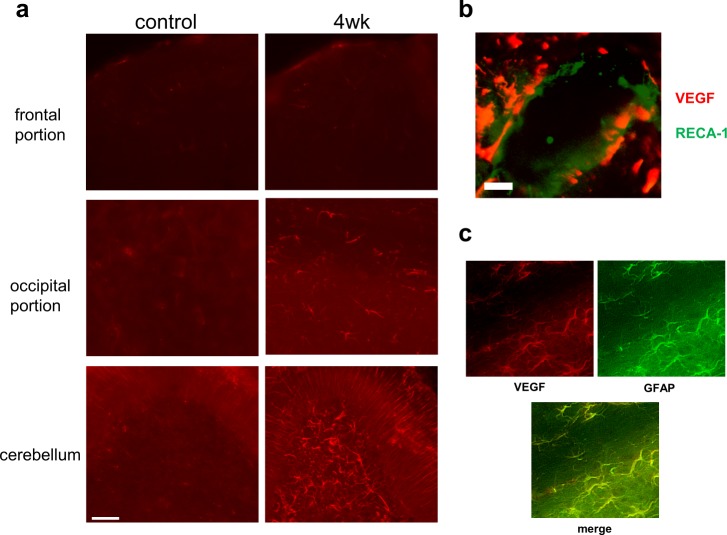
Vascular endothelial growth factor (VEGF) expression associated with methylmercury exposure. (a) Immunohistochemical staining was performed using rabbit anti-VEGF antibody to detect VEGF expression in the frontal and occipital regions and cerebellum of rats in the control and 4-week MeHg exposure groups (left and right panels, respectively). Scale bar, 50 μm. (b) Representative images of double immunohistochemical staining sections of cerebellum in the 4-week MeHg exposure group. VEGF (red) and rat endothelial cell antigen-1 (RECA-1, a marker of endothelial cells, green) positive cells are shown. Scale bar, 20 μm. (c) Double immunohistochemical staining for VEGF (red) and glial fibrillary acidic protein (GFAP, a marker of astrocytes, green). Scale bar, 30 μm. All experiments were performed in triplicate.

To investigate whether subacute MeHg intoxication increases VEGF expression in the central nervous system, we performed IHC staining of the cerebrum and cerebellum using an antibody against VEGF. VEGF expression in the cerebellum markedly increased in the 4-week exposure group when compared with that of the control group (Fig 5A), although there was no apparent VEGF expression until 3 weeks of exposure (data not shown). VEGF expression was detected in both the cerebellar cortex and medulla, which was evident in the granular cell layer. VEGF expression increased mildly in the occipital region. In contrast, no significant increase in VEGF expression was observed in the frontal and central regions. We also investigated the cellular localization of VEGF using antibodies against the astrocyte marker GFAP and endothelial cell marker RECA-1. VEGF was expressed on the outer side of the RECA-1-positive endothelial cells (Fig 5B), and most of the VEGF-expressing cells were GFAP-positive astrocytes (Fig 5C).

### BBB damage in rats exposed to MeHg

**Fig 6:**
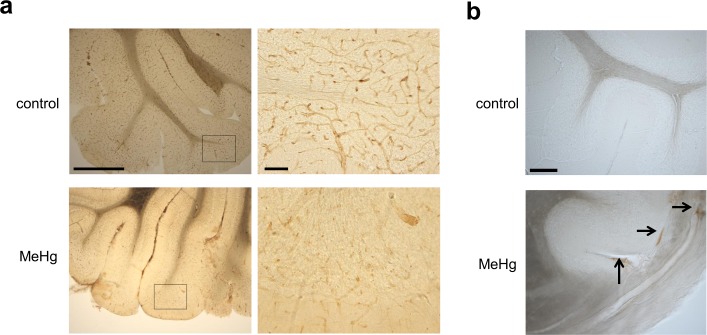
Expression of endothelial cell markers and IgG extravasation in the cerebellum of rats exposed to methylmercury. (a) Rat cerebellum sections from control and 4-week exposure groups (upper and lower panels, respectively) were stained using an antibody against rat endothelial cell antigen-1 (RECA-1). Low and high (left and right panel, respectively) magnification images are shown. Scale bars, 100 and 10 μm (left and right panel, respectively). (b) Rat cerebellum sections from control and 4-week exposure groups (upper and lower panels, respectively) were stained using an antibody against rat IgG. Vascular hyperpermeability was evaluated by immunostaining of intrinsic IgG outside of vessels in control and 4-week exposure groups. Arrows indicate IgG extravasation in the 4-week MeHg exposure group. No IgG staining outside of vessels was detected in the control group. Scale bar, 25 μm. All experiments were performed in triplicate.

We investigated whether MeHg exposure resulted in BBB damage and vascular hyperpermeability using sections from the rat cerebellum. IHC staining using an antibody against RECA-1 demonstrated a decrease in RECA-1 expression in the cerebellum of rats exposed to MeHg for 4 weeks relative to unexposed controls (Fig 6A), although no obvious cellular damage was observed until 3 weeks of exposure. Extravasation of endogenous IgG revealed positive immunostaining outside of the vessels of the cerebellum only in the rats exposed to MeHg for 4 weeks (Fig 6B).

### Effect of anti-VEGF neutralizing antibody on hind-limb crossing sign

**Fig 7:**
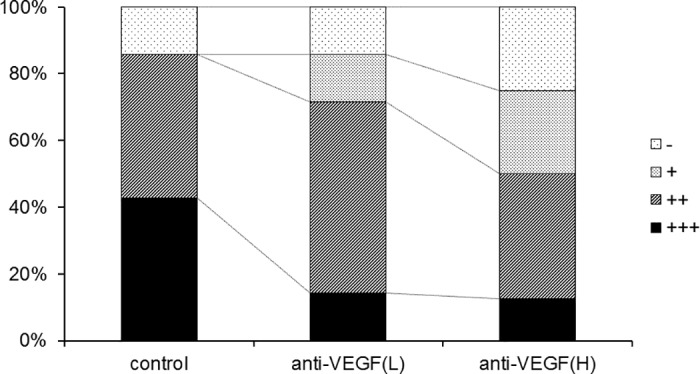
Hind-limb crossing signs after 4-week exposure to methylmercury (MeHg) with or without intravenous administration of anti-vascular endothelial growth factor (VEGF) neutralizing antibody. Neuronal impairment was evaluated by assessing the presence of hind-limb crossing signs after 4 weeks of MeHg exposure with or without anti-VEGF antibody treatment. Neuronal impairment was evaluated according to the following scale: (+++) limbs crossed each other; (++), limbs nearly crossed; (+) limbs did not cross and limb flexion was observed; and (-), limbs moved freely and were typically splayed outward; n = six to eight per group. Control, MeHg exposure without treatment; anti-VEGF (L), low dose anti-VEGF antibody group; anti-VEGF (H), high dose anti-VEGF group.

Finally, we investigated the effect of anti-VEGF neutralizing antibody on hind-limb crossing signs at 10 weeks of age (Fig 7). The rate of complete crossing (+++) was reduced from 43% in the control group to 14% and 13% in the VEGF (L) and VEGF (H) groups, respectively.

## Discussion

Using a rat model of subacute MeHg intoxication, we demonstrated for the first time that MeHg causes BBB damage *in vivo*. The BBB damage in the cerebellum was confirmed by extravasation of endogenous IgG and decreased expression of RECA-1. In addition, we observed increased expression of VEGF in the cerebellum and occipital lobe in rats exposed to MeHg. VEGF expression was detected in rats exposed to MeHg for 4 weeks mainly in astrocytes, major components of the BBB. These findings are consistent with the observation that edema and hemorrhage are observed in the cerebellum of patients with severe cases of Minamata disease exhibit [20],[21].

Elucidating the mechanism of VEGF upregulation in astrocytes is important for understanding the pathophysiological nature of MeHg toxicity. Some researchers have suggested that VEGF expression may be upregulated by the transcriptional factor hypoxia inducible factor (HIF)-1α. Reactive oxygen species (ROS) are generated by MeHg *in vitro* [22] and *in vivo* [23]. ROS have been implicated in the regulation of hypoxic and non-hypoxic induction of HIF-1α under various conditions, including MeHg intoxication [24]. Furthermore, research has indicated that HIF-1α directly upregulates transcription of VEGF [24]. Therefore, it is possible that ROS induced by MeHg results in VEGF upregulation via HIF-1α. Alternatively, VEGF expression may be induced by MeHg via inhibition of aquaporin (AQP) 4 water channels, as mercury is a strong inhibitor of AQP4. Indeed, previous studies have demonstrated that AQP4 inhibition in adaptive astrocytes of the retina known as Müller cells induces VEGF upregulation [25].

The results of the present study also indicate that MeHg may produce BBB damage. Researchers of our group, as well as others, have reported that the cerebellum and dorsal root ganglion are the most severely injured sites in rat models of MeHg intoxication [9],[26]. The dorsal root ganglion is not covered by the BBB, and the BBB of the cerebellum is thought to be more vulnerable to MeHg-induced toxicity than that of the cerebrum [27]. These findings suggest that neuronal damage might occur at regions where the barrier function of BBB is lacking or relatively weak, and that the BBB may protect against neuronal damage associated with MeHg. In several diseases such as ischemic stroke, viral encephalitis, and traumatic brain injury, research has demonstrated that neuronal damage is exacerbated by BBB damage via non-selective influx of cytotoxic agents or inflammatory cells from the blood into the brain tissue [28],[29],[30]. Based on these findings, we speculate that BBB damage associated with VEGF expression facilitates neuronal damage following exposure to MeHg. Therefore, VEGF may be a potential therapeutic target for the treatment of MeHg intoxication.

In the present study, we also observed that the effect of an antibody against VEGF on neuronal impairment as assessed by hind-limb crossing signs was limited. However, there are two possible explanations for this result. It is possible that MeHg exerts neurotoxicity via multiple mechanisms. Alternatively, assessment of hind-limb crossing signs is not necessarily an appropriate method for evaluating the effect of antibodies against VEGF, as such signs reflect not only cerebellar ataxia but also disturbances of deep sensation related to the degeneration of the dorsal root ganglion. Thus, future studies are preferable to determine effects of combined therapy using a chelating drug/free radical scavenger and vascular protective drugs using more appropriate testing methods. Moreover, adding another functional test such as the rotarod test will be helpful in more sensitive evaluation although such tests may not specifically reflect cerebellar dysfunction.

In conclusion, we demonstrated for the first time that MeHg induces VEGF upregulation in the cerebellum as well as BBB damage *in vivo*. Inhibition of VEGF aimed at protecting the BBB may represent a promising therapeutic strategy for the treatment of MeHg intoxication.

## References

[1] Tokuomi H, Okajima T, Kanai J, Tsunoda M, Ichiyasu Y, Misumi H et al Minamata disease—an unusual neurological disorder occurring in Minamata, Japan. Kumamoto Med J 1961 14:47–64

[2] Takizawa Y. Studies on the Niigata episode of Minamata disease outbreak. Investigation of causative agents of organic mercury poisoning in the district along the river Agano. Acta Med Biol (Niigata). 1970 17(4):293–7. 5464506

[3] Streets DG, Devane MK, Lu Z, Bond TC, Sunderland EM, Jacob DJ. All-time releases of mercury to the atmosphere from human activities. Environ Sci Technol 2011 45:10485–91. 10.1021/es202765m 22070723 PMC3246392

[4] Hunter D, Russell DS. Focal cerebellar and cerebellar atrophy in a human subject due to organic mercury compounds. J Neurol Neurosurg Psychiatry. 1954 17(4):235–241. 13212411 10.1136/jnnp.17.4.235 PMC503192

[5] Takeuchi T. Pathology of Minamata disease. With special reference to its pathogenesis. Acta Pathol Jpn 1982 32 Suppl 1:73–99. 6765001

[6] Eto K. Pathology of Minamata disease. Toxicol Pathol 1997 25:614–23. 9437807 10.1177/019262339702500612

[7] Shirakawa K. Clinical and epidemiological study on Minamata disease in Niigata. Seishin Shinkeigaku Zasshi. 1972 8;74(8):679–89. Japanese. 4675937

[8] Kojima K, Fujita M. Summary of recent studies in Japan on methyl mercury poisoning. Toxicology. 1973 3;1(1):43–62. 4203273 10.1016/0300-483x(73)90017-6

[9] Usuki F, Yasutake A, Umehara F, Tokunaga H, Matsumoto M, Eto K et al In vivo protection of a water-soluble derivative of vitamin E, Trolox, against methylmercury-intoxication in the rat. Neurosci Lett 2001 304:199–203. 11343836 10.1016/s0304-3940(01)01764-5

[10] Farina M, Campos F, Vendrell I, Berenguer J, Barzi M, Pons S et al Probucol increases glutathione peroxidase-1 activity and displays long-lasting protection against methylmercury toxicity in cerebellar granule cells. Toxicol Sci 2009 112:416–26. 10.1093/toxsci/kfp219 19770487

[11] Fujimura M, Usuki F. Low in situ expression of antioxidative enzymes in rat cerebellar granular cells susceptible to methylmercury. Arch Toxicol 2014 88:109–13. 10.1007/s00204-013-1089-2 23832298

[12] Hirooka T, Yamamoto C, Yasutake A, Eto K, Kaji T. Expression of VEGF-related proteins in cultured human brain microvascular endothelial cells and pericytes after exposure to methylmercury. J Toxicol Sci 2013 38:837–45. 24213003 10.2131/jts.38.837

[13] Ferrara N, Gerber H-PP, LeCouter J. The biology of VEGF and its receptors. Nat Med 2003 9:669–76. 10.1038/nm0603-669 12778165

[14] Fujimura M, Usuki F, Kawamura M, Izumo S. Inhibition of the Rho/ROCK pathway prevents neuronal degeneration in vitro and in vivo following methylmercury exposure. Toxicol Appl Pharmacol 2011 250:1–9. 10.1016/j.taap.2010.09.011 20869980

[15] Jacobs MB, Yamaguchi S, Goldwater LJ, Gilbert H. Determination of mercury in blood. Am Ind Hyg Assoc J 1960 21:475–80. 10.1080/00028896009344108 13718571

[16] Moser VC, Screening approaches to neurotoxicity: a functional observational battery. J. Am. Coll. Toxicol. 1989 8;85–93.

[17] Chakrabarti SK, Bai C. Effects of protein-deficient nutrition during rat pregnancy and development on developmental hindlimb crossing due to methylmercury intoxication. Arch Toxicol 2000 74:196–202. 10959792 10.1007/s002040000112

[18] Kimura R, Nakase H, Tamaki R, Sakaki T. Vascular endothelial growth factor antagonist reduces brain edema formation and venous infarction. Stroke 2005 36:1259–63 10.1161/01.STR.0000165925.20413.14 15879344

[19] Kanazawa M, Igarashi H, Kawamura K, Takahashi T, Kakita A, Takahashi H et al Inhibition of VEGF signaling pathway attenuates hemorrhage after tPA treatment. J Cereb Blood Flow Metab 2011 31:1461–74. 10.1038/jcbfm.2011.9 21304556 PMC3130331

[20] Eto K, Takizawa Y, Akagi H, Haraguchi K, Asano S, Takahata N et al Differential diagnosis between organic and inorganic mercury poisoning in human cases—the pathologic point of view. Toxicol Pathol 1999 27:664–71. 10588547 10.1177/019262339902700608

[21] Philbert Martin A., Billingsley Melvin L. and Reuhl Kenneth R. Mechanisms of Injury in the Central Nervous System. Toxicol Pathol. 2000 28:43–53; 10668990 10.1177/019262330002800107

[22] Hagele TJ, Mazerik JN, Gregory A, Kaufman B, Magalang U, Kuppusamy ML et al Mercury activates vascular endothelial cell phospholipase D through thiols and oxidative stress. J Biol Chem. 2011;286(8):6641–9. 17365148 10.1080/10915810601120509

[23] Usuki F, Yamashita A, Fujimura M. Post-transcriptional defects of antioxidant selenoenzymes cause oxidative stress under methylmercury exposure. J Biol Chem. 2011;286(8):6641–9. 10.1074/jbc.M110.168872 21106535 PMC3057802

[24] Ema M, Taya S, Yokotani N, Sogawa K, Matsuda Y, Fujii-Kuriyama Y. A novel bHLH-PAS factor with close sequence similarity to hypoxia-inducible factor 1α regulates VEGF expression and is potentially involved in lung and vascular development. Proc Natl Acad Sci USA. 1997;94:4273–4278 9113979 10.1073/pnas.94.9.4273 PMC20712

[25] Cui B, Sun JH, Xiang FF, Liu L, Li WJ. Aquaporin 4 knockdown exacerbates streptozotocin-induced diabetic retinopathy through aggravating inflammatory response. Exp Eye Res. 2012 5;98:37–43. 10.1016/j.exer.2012.02.013 22449442

[26] Wakabayashi K, Kakita A, Sakamoto M, Su M, Iwanaga K, Ikuta F. Variability of brain lesions in rats administered methylmercury at various postnatal development phases. Brain Res 1995 705:267–72. 8821758 10.1016/0006-8993(95)01208-7

[27] Silwedel C, Förster C. Differential susceptibility of cerebral and cerebellar murine brain microvascular endothelial cells to loss of barrier properties in response to inflammatory stimuli. J Neuroimmunol. 2006 179(1–2):37–45. 10.1016/j.jneuroim.2006.06.019 16884785

[28] Rosenberg GA, Estrada EY, Dencoff JE. Matrix metalloproteinases and TIMPs are associated with blood-brain barrier opening after reperfusion in rat brain. Stroke 1998 29(10):2189–95. 9756602 10.1161/01.str.29.10.2189

[29] Zhou J, Stohlman SA, Hinton DR, Marten NW. Neutrophils promote mononuclear cell infiltration during viral-induced encephalitis. J Immuno. 2003 170(6):3331–6. 10.4049/jimmunol.170.6.3331 12626593

[30] Sandoval KE, Witt KA. Blood-brain barrier tight junction permeability and ischemic stroke. Neurobiol Dis. 2008 32(2):200–19. 10.1016/j.nbd.2008.08.005 18790057

